# Sudden cardiac death in COVID-19 patients, a report of three cases

**DOI:** 10.2217/fca-2020-0082

**Published:** 2020-07-03

**Authors:** Samira Shirazi, Sanaz Mami, Negar Mohtadi, Abas Ghaysouri, Hamed Tavan, Ali Nazari, Taleb Kokhazadeh, Reza Mollazadeh

**Affiliations:** ^1^Assistant Professor of Cardiology, Department of Cardiology, School of Medicine, Shahid Mostafa Khomeini Hospital, Ilam University of Medical sciences, Ilam, Iran; ^2^Assistant Professor of Medical Immunology, Department of Immunology, School of Medicine, Ilam University of Medical Sciences, Ilam, Iran; ^3^Assistant Professor of Infectious Disease, School of Medicine, Shahid Mostafa Khomeini Hospital, Ilam University of Medical Sciences, Ilam, Iran; ^4^Assistant Professor of Pulmonary Diseases, Department of Internal Medicine, School of Medicine, Ilam University of Medical Sciences, Ilam, Iran; ^5^MSC of Medical Surgical Nursing, Clinical Research Development Unit, Shahid Mostafa Khomeini Hospital, Ilam University of Medical Sciences, Ilam, Iran; ^6^MSC of Critical care Nursing, Clinical Research Development Unit, Shahid Mostafa Khomeini Hospital, Ilam University of Medical Sciences, Ilam, Iran; ^7^Associate Professor of Cardiology, Department of Cardiology, Imam Khomeini Hospital Complex, Tehran University of Medical Sciences, Tehran, Iran

**Keywords:** arrhythmia, cardiac, coronavirus, COVID-19, sudden cardiac death

## Abstract

The mortality rate of coronavirus disease-19 (COVID-19) has been reported as 1–6% in most studies. The cause of most deaths has been acute pneumonia. Nevertheless, it has been noted that cardiovascular failure can also lead to death. Three COVID-19 patients were diagnosed based on reverse transcriptase-polymerase chain reaction of a nasopharyngeal swab test and radiological examinations in our hospital. The patients received medications at the discretion of the treating physician. In this case series, chest computed tomography scans and electrocardiograms, along with other diagnostic tests were used to evaluate these individuals. Sudden cardiac death in COVID-19 patients is not common, but it is a major concern. So, it is recommended to monitor cardiac condition in selected patients with COVID-19.

Within less than six months, COVID-19 has now spread from a market in Wuhan, China, across more than 150 countries and transformed to a pandemic [1]. The infection presents with symptoms such as fever, cough, fatigue, sputum, muscle ache, dyspnea and eventually severe acute respiratory failure [1,2].

There is not much information about the mortality rate, which varies in different countries. Some studies have reported mortality rates of 1–2% [2,3]. In comparison, severe acute respiratory syndrome coronavirus (SARS-CoV) and Middle East respiratory syndrome-related coronavirus (MERS-CoV), viruses of this family, had mortality rates of 10–35% [4]. In a retrospective study by Chen *et al.* [5] in January 2020, out of 99 patients with COVID-19, 57 (58%) were hospitalized, 31 (31%) were discharged and only 11 (11%) died of the infection. Also, in another study conducted in Iran, the mortality rate was reported to be 7.14% [6].

According to studies, the main cause of death in COVID-19 patients is severe pneumonia. However, it has been reported that mortality in the patients is significantly associated with pre-existing cardiovascular conditions [7]. In another study, Italian researchers highlighted the role of hypertension in increasing COVID-19-related mortality as high as 2.5-times in this subgroup [8]. Although clinical manifestations of COVID-19 infection are unknown in patients with cardiac conditions, evidence obtained from patients with end-stage heart failure indicates that the virus can inflict or exaggerate cardiac damage [9].

Recently, an Italian group reported that during the COVID-19 outbreak in 2020 a 58% increase in out-of-hospital cardiac arrest cases occurred when compared with the same period in 2019. Notably, the cumulative incidence of out-of-hospital cardiac arrest in 2020 strongly associated with the cumulative incidence of COVID-19. Moreover, they estimated that patients receiving a COVID-19 diagnosis accounted for most of such an increase of events, close to 80% of cases [10].

In the present study, we reported sudden cardiac death, which is not the dominant mode of death, in three patients with COVID-19 infection admitted to Shahid Mostafa Khomeini Hospital of Ilam in March and April 2020.

## Case presentation

### Case 1

**Figure 1.**
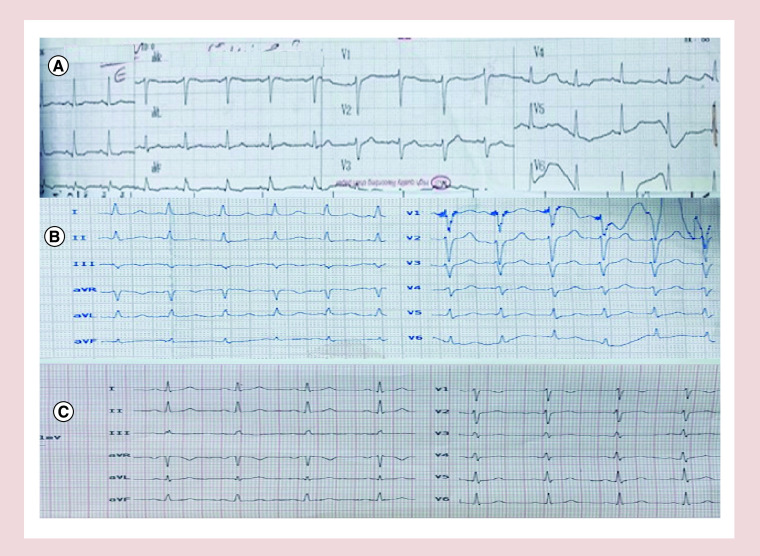
ECG of the patients. **(A)** ECG shows normal sinus rhythm, normal axis, ST segment depression and T wave inversion in II, III, aVF, QT and QT_C_ intervals are 380 and 480 msec, respectively. **(B)** ECG showed normal sinus rhythm, normal axis, QT and QT_C_ intervals are 400 and 500 msec, respectively. **(C)** Normal sinus rhythm, normal axis, QT and QT_C_ intervals are 380 and 425 msec, respectively.

**Figure 2.**
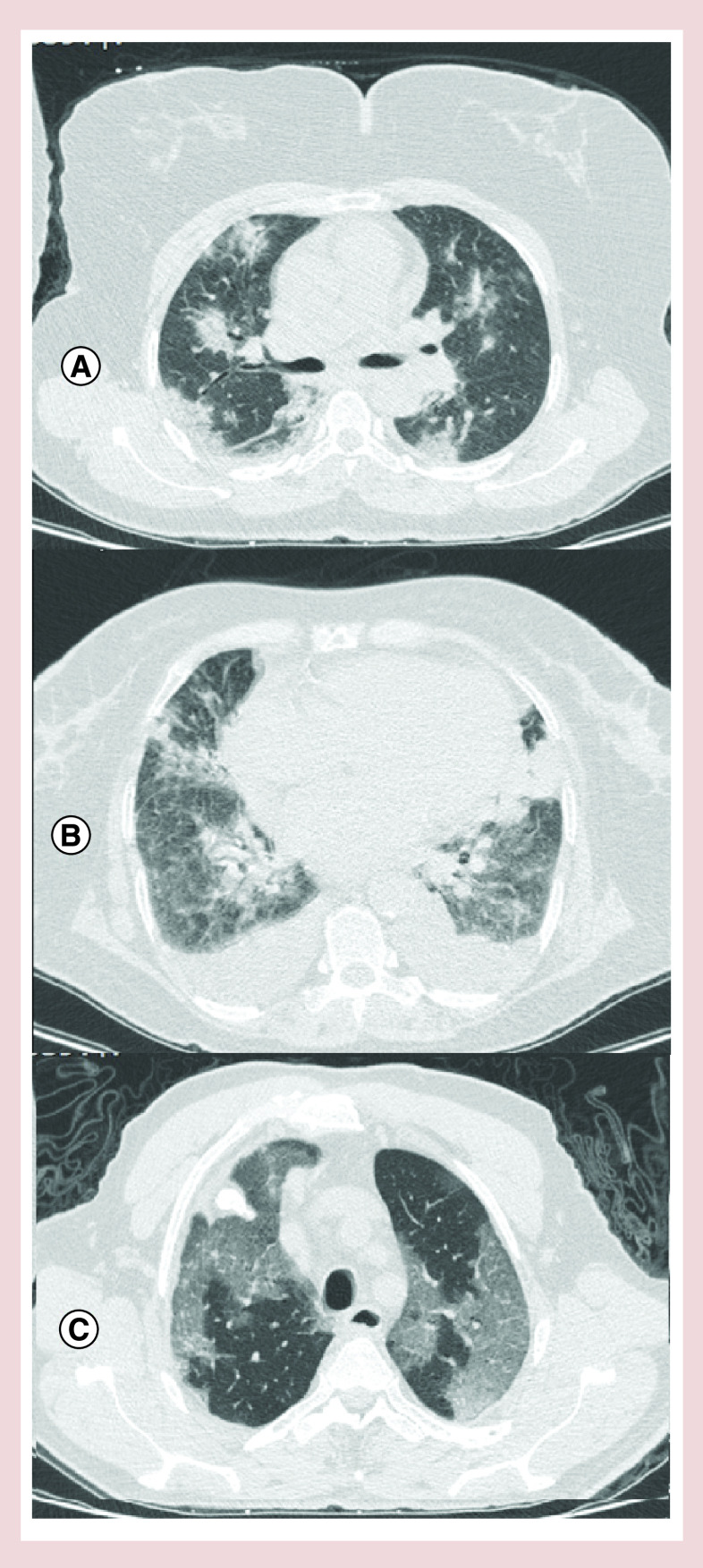
Chest high-resolution computed tomography of the patients. **(A)** Lung high-resolution computed tomography showed multifocal peripheral ground glass opacity in both lungs, highly in favor of COVID-19 pneumonia. **(B)** Axial thin-section noncontrast CT scan shows diffuse bilateral confluent and patchy ground-glass and consolidative pulmonary opacities with bilateral pleural effusion. **(C)** Chest CT scan image shows multiple patchy, peripheral, bilateral areas of ground-glass opacities.

A 50-year-old woman without known history of specific diseases or using specific drugs, complaining of fever, chills and dry cough was hospitalized in our center. On admission, her condition was as blood pressure (BP) = 130/80 mmHg, pulse rate (PR) = 80/min, body temperature (BT) = 37.2°C and O_2_ saturation (in room air) = 95%. She had no abnormal findings at the initial examination. Baseline ECG was within normal limits (Figure 1A). Due to pulmonary involvement evidenced in chest CT scan (Figure 2A), she was treated with levofloxacin, vancomycin, hydroxychloroquine, lopinavir/ritonavir, as well as heparin for prophylaxis of deep venous thrombosis at standard doses. There was no electrolyte abnormality during hospitalization. On the fifth day of hospitalization, the treating physician decided to discharge the patient and continue the rest of treatment at home. While being discharged; the patient developed a sudden cardiac arrest and died as resuscitation was ineffective. The patient’s laboratory information has been shown in Table 1.

**Table 1.** Laboratory information of three patients’ with COVID-19 infection who died of sudden cardiac arrest.

Laboratory variables	First case	Second case	Third case
WBC (*10^9^/l)	3.4	9.1	16
RBC (*10^6^ cells/mcl)	4.26	2.9	4.56
Hemoglobin (g/dl)	12.6	9	14.4
Hematocrit (%)	37.8	25.1	43
MCV (fl)	89	86	94
MCH (pg)	29.5	29	31.6
MCHC (g/dl)	33.3	33.5	33.5
ESR (mm/h)	31	125	82
Platelets (*10^9^/l)	158	382	147
Neutrophil (*10^9^/l)	75	82	90
Lymphocyte (*10^9^/l)	20	18	10
Monocyte (*10^9^/l)	2	N/A	N/A
CRP (Qualitative)	2+	3+	3+
AST (U/l)	N/A	28	N/A
ALT (U/l)	N/A	16	N/A
Alkaline phosphatase (IU/l)	N/A	187	N/A
Blood sugar (mg/dl)	75	N/A	N/A
Blood urea (mg/dl)	26	77	25
Serum creatinine	1.1	1.5	1
Blood sodium (mmol/l)	138	133	142
Blood potassium (mmol/l)	4	4	4.3
Serum phosphate (mg/dl)	2.6	3.4	3.4
Serum magnesium (mg/dl)	2.7	2.6	2.06
Serum calcium (mg/dl)	10.2	9.6	8.6

Note: The symbol * denotes multiplication in the table.

ALT: Alanine aminotransferase; AST: Aspartate aminotransferase; CRP: C-reactive protein; ESR: Erythrocyte sedimentation rate; MCH: Mean corpuscular hemoglobin; MCHC: Mean corpuscular hemoglobin concentration; MCV: Mean corpuscular volume; N/A: Not available; RBC: Red blood cell; WBC: White blood cell.

### Case 2

The patient was a 75-year-old woman with a history of diabetes and chronic renal failure hospitalized due to dyspnea. The vital signs at the time of referral were as BP = 100/75, PR = 105/min, RR: 18/min, BT: 37 and O_2_ saturation (in room air) = 87%. Crackles were heard in both lungs while other examinations were normal. Due to pulmonary involvement and dyspnea, the patient was treated with oseltamivir, hydroxychloroquine, lopinavir/ritonavir, meropenem, dexamethasone and enoxaparin adjusted based on serum creatinine level. The patient was consulted with a cardiologist and an endocrinologist. ECG (Figure 1B) and echocardiography revealed no abnormality and the blood glucose level was closely monitored. According to recommendations, the patients was treated with corticosteroids as well. The result of troponin I test was negative and creatinine level was reported as 1.5 mg/dl. There were no electrolyte abnormalities during hospitalization. After 7 days and with the improvement of symptoms and reaching O_2_ saturation >95% within the last 48 h, the patient was decided to continue quarantine in a recovery center. The next day, after being transferred while having good general condition and normal vital signs, she suddenly developed cardiac arrest and died due to lack of response to resuscitation. Table 1 shows the patient’s laboratory information and Figure 2B demonstrates the results of the patient’s chest CT scan.

### Case 3

A 60-year-old man without any history of specific diseases was referred to our hospital due to fever and dyspnea. He was diagnosed with COVID-19 and hospitalized. On admission, the patient's vital signs were as BP = 110/70, PR = 105/min, BT = 38.7°C and O_2_ saturation (in room air) = 82% which increased to >94% after applying O_2_ masks. During a clinical examination, crackle was heard in the patient’s left lung. He was treated with oseltamivir, hydroxychloroquine, lopinavir/ritonavir and azithromycin. The patient had no specific problems and the vital signs were normal with no evidence of electrolyte disturbances. The troponin I test was negative. Baseline ECG was normal (Figure 1C). On day 2 of hospitalization, he suffered from cardiac arrest and unfortunately died as resuscitation was unsuccessful. Table 1 shows the patient’s laboratory information; and the results of the patient’s chest CT scan is shown in Figure 2C.

## Discussion

During the past 18 years, coronaviruses have caused three major crises in human societies [4]. For the first time, in November 2002, SARS emerged due to SARS-CoV and spread across China. Then in September 2012, Middle East respiratory syndrome caused by the MERS-CoV spread in many parts of the world causing many deaths. And now, there has been a new pneumonia related to COVID-19 virus identified in December 2019 turning to a global pandemic since March 2020 [5]. Because of being highly contagious, as well as having high mortality rate in the elderly and people with pre-existing medical conditions [11], many concerns have been raised worldwide regarding the pandemic global outcomes.

Many studies have reported pneumonia and acute respiratory distress as the main causes of death in COVID-19 patients. Underlying diseases that increase the risk of mortality due to COVID-19 include high BP, cardiovascular and cerebral disease, diabetes, hyperlipidemia, peripheral vascular diseases and chronic renal failure [11]. Numerous studies have highlighted an association between cardiovascular conditions and risk of mortality in COVID-19 patients [7]. Cardiac troponin I is one of the laboratory parameters predicting cardiac ischemia in patients with COVID-19. In fact, this laboratory parameter indicates cardiac damage in the patients [12]. Although most studies have noted that cardiac damage as a risk factor of mortality, Inciardi *et al.* reported death due to cardiac failure in a patient with COVID-19 who had no history of cardiovascular problems. However, there was no indication of possible mechanisms of cardiac failure in this recent report [13].

Viral infection is an important cause of myocarditis. The most well-known viruses of this type include influenza and parvovirus B-19. Nevertheless, it is not clear whether SARS-CoV-2 also induces cardiac damage. Possible mechanisms by which COVID-19 may cause cardiac damage include inflammatory responses and cytokine storm, direct attack to cardiomyocytes and inducing severe hypoxia.

Another potential cause is the proarrhythmic effects of hydroxychloroquine. This is the standard of treatment for COVID-19 in our country, Iran. QT prolongation and torsades de pointes (TdP) are a known adverse effect of this drug. Besides hydroxychloroquine, other risky drugs include lopinavir/ritonavir (administered in all 3 cases), azithromycin (case 3) and levofloxacin (case 1) may lead to (TdP) [14]. Although we did not have the ECG of the patients prior to their death to prove this theory.

Another potentially important factor involved in QT_C_ prolongation in COVID-19 is the high-grade systemic inflammation which characterize the diseases, frequently a real ‘cytokine storm’ in which IL-6 seems to play a pivotal role. As recently pointed out by PE *et al.*, IL-6 could promote QT_C_ prolongation in COVID-19 patients by different mechanisms [15].

Another very rare cause could be transient bradycardia in these patients. We have reported this complication before [16].

In compliance with our national guidelines, all patients admitted with the diagnosis of COVID-19, should be treated with hydroxychloroquine and ECG is obtained for patients >40 years old or with known cardiovascular disease. It is recommended to repeat the ECG on the third to fifth day of therapy. Prolongation of QT_C_ intervals in the first and second case (480 and 500 msec, respectively) could be due to inflammatory activation (besides pre-existing risk factors, specifically diabetes and chronic heart failure in patient 2). Thus, it is plausible that a further QT_C_ increase occurred after starting drug assumption, possibly reaching critical levels to favor TdP and SCD.

According to the official reports by the Shahid Mostafa Khomeini Hospital of Ilam, 353 patients have been confirmed with COVID-19 infection until 10 April 2020 in Ilam Province. Of these, 35 deaths have been reported giving a mortality rate of 9.91%. From the 35 deceased cases, sudden cardiac arrest had been observed in three patients rendering an uncommon condition (0.84%). These patients had no symptoms or recognizable predictors requiring more examinations to timely identify them.

### Study limitations

ECG of the patients prior to their death are not available and in that case we could discuss more precisely about the QT and QT_C_.

## Conclusion

Death due to sudden cardiac arrest is not common, but possible, in COVID-19 patients. It is recommended to examine and monitor COVID-19 patients’ cardiac condition to identify at risk individuals.

**Summary points**

- COVID-19 pandemic is growing day by day.
- Although the cardinal manifestations are pulmonary, cardiovascular involvements are illustrated in the literature.
- Sudden cardiac arrest and death may happen in COVID-19.
- Herein we reported three sudden cardiac deaths among 353 proved COVID-19 patients in our institution.
- Interestingly, death occurred despite improvement of general condition and constitutional symptoms.
- Sudden death could be due direct involvement of myocardium by virus and arrhythmic events, cytokine storm or adverse drug effects (hydroxychloroquine and antivirals).
